# Quantifying brain volumes for Multiple Sclerosis patients follow‐up in clinical practice – comparison of 1.5 and 3 Tesla magnetic resonance imaging

**DOI:** 10.1002/brb3.422

**Published:** 2016-01-12

**Authors:** Andreas P. Lysandropoulos, Julie Absil, Thierry Metens, Nicolas Mavroudakis, François Guisset, Eline Van Vlierberghe, Dirk Smeets, Philippe David, Anke Maertens, Wim Van Hecke

**Affiliations:** ^1^ Department of NeurologyHôpital ErasmeUniversité Libre de BruxellesAnderlechtBelgium; ^2^ Department of RadiologyHôpital ErasmeUniversité Libre de BruxellesAnderlechtBelgium; ^3^ IcometrixLeuvenBelgium

**Keywords:** Brain atrophy, brain volume, MRI, MSmetrix, Multiple Sclerosis

## Abstract

**Introduction:**

There is emerging evidence that brain atrophy is a part of the pathophysiology of Multiple Sclerosis (MS) and correlates with several clinical outcomes of the disease, both physical and cognitive. Consequently, brain atrophy is becoming an important parameter in patients' follow‐up. Since in clinical practice both 1.5Tesla (T) and 3T magnetic resonance imaging (MRI) systems are used for MS patients follow‐up, questions arise regarding compatibility and a possible need for standardization.

**Methods:**

Therefore, in this study 18 MS patients were scanned on the same day on a 1.5T and a 3T scanner. For each scanner, a 3D T1 and a 3D FLAIR were acquired. As no atrophy is expected within 1 day, these datasets can be used to evaluate the median percentage error of the brain volume measurement for gray matter (GM) volume and parenchymal volume (PV) between 1.5T and 3T scanners. The results are obtained with MSmetrix, which is developed especially for use in the MS clinical care path, and compared to Siena (FSL), a widely used software for research purposes.

**Results:**

The MSmetrix median percentage error of the brain volume measurement between a 1.5T and a 3T scanner is 0.52% for GM and 0.35% for PV. For Siena this error equals 2.99%. When data of the same scanner are compared, the error is in the order of 0.06–0.08% for both MSmetrix and Siena.

**Conclusions:**

MSmetrix appears robust on both the 1.5T and 3T systems and the measurement error becomes an order of magnitude higher between scanners with different field strength.

## Introduction

Brain atrophy is a global marker of neuro‐axonal loss resulting from demyelination and neuronal pathology (Giorgio et al. 2008; Filippi and Agosta 2010). It is now known that brain atrophy occurs in all clinical stages of Multiple Sclerosis (MS) at a rate of 0.5–1.0%/year versus 0.1–0.3%/year in healthy subjects (Giorgio et al. 2008; Filippi and Agosta 2010).

Different hypotheses have been addressed to explain atrophy in MS: dysfunction in neuronal connectivity, anterograde transynaptic degeneration, retrograde degeneration, wallerian degeneration or neuronal soma, and dendritic shrinkage (Siffrin et al. 2010).

Brain atrophy is generally measured on 2D/3D T1‐weighted images and it is analyzed using cross‐sectional methods comparing patients to controls [e.g., brain parenchymal fraction (BPF), Structural Image Evaluation, using Normalization, of Atrophy (SIENAX), voxel‐based morphometry (VBM)] as well as longitudinal methods (e.g., SIENA)) (Giorgio et al. 2008).

Focal white matter (WM) lesions are the classic hallmark of MS. Profound alterations in normal‐appearing WM (NAWM) and gray matter (GM) are associated with progressive loss of brain volume (Markovic‐Plese and McFarland 2001; Smirniotopoulos et al. 2007; Kutzelnigg and Lassmann 2014). As a result, brain volume loss in MS occurs in both GM and WM (Filippi et al. 2012) in early and during all disease stages and subtypes (Giorgio and De Stefano 2010). In addition, it has been demonstrated that brain volume loss is a predictor of long‐term disability progression (Popescu et al. 2013) and a marker of cognitive decline in MS (Christodoulou et al. 2003; Morgen et al. 2006; Amato et al. 2007; Houtchens et al. 2007). Therefore, brain volume evolution is emerging as one of the four parameters of MS to be considered when evaluating disease activity (NEDA‐4 (no evidence of disease activity: relapses, EDSS, T2/Gd lesions, brain volume) (Giovannoni et al. 2015).

As brain atrophy is related to clinical outcomes in MS, there is need for brain atrophy analysis on individual subjects in order to monitor treatment efficacy. However, in order to use brain atrophy measures in clinical practice, it is of paramount importance that the measurement error is very small. As the whole‐brain atrophy rate in MS patients is in the order of 0.5–1%, reliable detection of subtle changes in brain volume is needed. MSmetrix brain volume measurements have been extensively tested for accuracy and precision in order to make it suitable for clinical practice. The method has obtained the CE mark and is approved for clinical use in Europe, Australia, India, Canada, Brazil, and Iran. An additional challenge for using automated measurements in clinical practice is that the methods should be robust among different scanner types.

In this manuscript, we assess the intra and interscanner variability in two methods for automated brain for automated brain volume measurements at 1.5T and 3T MRI estimation at 1.5T and 3T MRI. To demonstrate the potential use in clinical practice, the measurement error within these scanners and between the scanners is evaluated. To this end, MS patients were scanned twice on both scanners during the same day.

## Materials and Methods

This prospective study was approved by our institutional review board and written informed consent was obtained from all participants (reference P2013/098**/**B406201316929).

### Patient population

Nineteen MS patients (12 Relapsing‐Remitting MS, six Secondary Progressive MS and one Primary Progressive MS) were enrolled. Inclusion criteria were MS diagnosis according to McDonald Criteria 2010 and no MRI contraindication. The mean age was 40 years old (range from 21 to 63 years old) and the female to male ratio 14:4. The mean EDSS was 3.1. The mean disease duration was 10 years. See Table 1 for the full overview of the population.

**Table 1:** Population overview with mean value, standard deviation and minimum and maximal values for age, disease duration since 1st symptoms, EDSS, brain volume, and lesion volume

	Age (years)	1st Symptoms (years)	EDSS	Lesion Volume (ml)	Whole‐Brain Volume (ml)	Gray Matter Volume (ml)
Mean	40	10	3.1	28.60	1021.40	637.91
SD	11	6	1.6	18.84	69.54	44.24
Min	21	3	1.5	2.64	891.75	560.63
Max	63	25	6.5	60.96	1134.88	704.42

### MRI protocol

The patients were scanned on two Philips Healthcare MR systems (Philips, Best, The Netherlands): Intera (1.5T) and Achieva (3T). On each scanner, a clinical MRI protocol was acquired, including a transverse 3D FLAIR (Fluid Attenuated Inversion Recovery) sequence and a sagittal 3D T1‐weighted turbo field echo sequence. The exact parameters are given in Table 2. This protocol was obtained twice on each scanner on the same day for all patients. Note that patients were not removed from the scanner in between the acquisition of the two MRI protocols.

**Table 2:** Scan protocol parameters for the 3D T1‐weighted sequence (upper) and the FLAIR sequence (lower) on the Intera (left) and Achieva (right) systems

	Intera	Achieva
3D T1 TFE		
Field strength	1.5 T	3.0 T
Acquisition voxel	0.87 × 1.25 × 1.2 mm³	0.88 × 1.19 × 1 mm³
FOV (field‐of‐view)	236 × 236 mm²	200 × 239 mm²
TR (repetition time)	8.8 msec	9.8 msec
TE (echo time)	4.2 msec	4.6 msec
Flip angle	8°	8°
Number of slices	130	160
Total scan duration	4 min 31 sec	5 min 35 sec
3D FLAIR		
Field strength	1.5 T	3.0 T
Acquisition voxel	1.36 × 1.77 × 1.5 mm³	1.31 × 1.34 × 1.3 mm³
FOV	240 × 192 mm²	230 × 167 mm²
TR	11000 msec	10000 msec
TE	140 msec	140 msec
TI (inversion delay)	2800 msec	2750 msec
Number of slices	96	105
Total scan duration	5 min 08 sec	7 min 30 sec

### Image analysis

Scanning the patient twice on each scanner, allows three different test–retest datasets to be analyzed. The first dataset includes for each patient two scan sessions on the Intera (1.5T), the second dataset is similar but all scans are acquired on the Achieva (3T) and the third dataset combines the first session from the Intera with the first session of the Achieva.

The different test–retest datasets, containing a 3D T1 and 3D FLAIR for two scan sessions on the same day, are analyzed with MSmetrix, a newly developed method to measure brain volume changes for MS patients.

**Figure 1:**
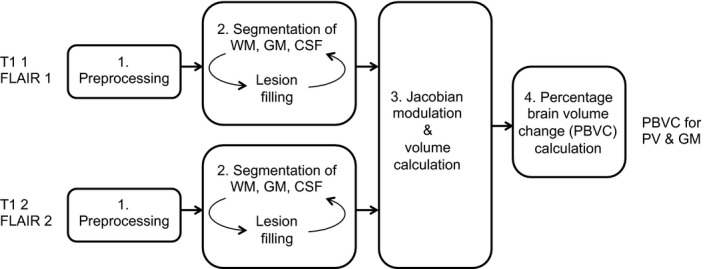
Schematic representation of MSmetrix, where the T1 and FLAIR of the two scan sessions that need to be compared are first preprocessed. Based on the preprocessed images, the segmentation of WM, GM and CSF is performed together with lesion filling in the second step. The third step is to calculate the volumes and perform the Jacobian modulation and only in the fourth step the actual PBVC is obtained.

MSmetrix is a CE approved automatic method for segmentation of GM, WM, cerebrospinal fluid (CSF) and white matter lesions based on unsupervised classification, as well as for a longitudinal atrophy measurement of whole brain or parenchymal volume (PV) and GM (Jain et al. 2015). It is an iterative method in order to optimize the segmentations of WM, GM, and CSF based on the WM lesion segmentation and vice versa until convergence of the results. Figure 1 shows a schematic overview of the method.

The first step is a preprocessing step, during which for each session the FLAIR image and the T1‐weighted image are rigidly coregistered to each other, followed by a skull‐stripping of the T1 image. In addition, probabilistic anatomical priors for WM, GM, and CSF are brought to the image space of the T1 image (Cardoso 2012).

In the second step, the segmentation of the different brain structures is carried out for each session, using an expectation‐maximization (EM) algorithm (Van Leemput et al. 2001) to model the intensities of each tissue class. In this step, also the white matter lesions are detected and filled so the lesion‐filled image can be segmented again. This iterative process is repeated until the results for WM, GM, CSF, and lesions do no longer change. Step 1 and 2 are still cross‐sectional, that is, the two scan sessions are processed separately.

In the third step, a jacobian modulation of the T1 images of each session to the T1 image of the other session provides us with a change in volume of one time point to the other. Now the information of both scan sessions is used together, which makes the method a longitudinal one.

In the last step, the volume changes of step three are averaged to obtain a robust measurement of the percentage brain volume change (PBVC) for PV and GM volume.

The results of MSmetrix are compared to the outcome obtained by SIENA (FSL, http://www.fmrib.ox.ac.uk/fsl), a commonly used software package for measuring whole‐brain atrophy (Smith et al. 2001a,b; Smith et al. 2002) First, the Brain Extraction Tool (BET) is applied, by making a histogram of intensities and transforming the image into a binary mask (Jenkinson and Smith 2001; Jenkinson et al. 2002). Subsequently, voxels within the obtained brain mask are classified in several classes, depending on the image intensities. As a result, CSF, WM, GM and background are segmented, and resulting cross‐sectional volumes can be obtained, referred to as SIENAX (González Ballester et al. 2000). Optimized brain extraction parameter settings were applied to ensure a correct masking of the brain (Popescu et al. 2012). A quality check was performed visually.

Based on the segmentation, brain parenchyma, or the combination of WM and GM, is classified and the edge between brain parenchyma and CSF is determined. When this is done for two MRI datasets of the same subject, they can be both transformed to an intermediate space using an affine transformation. Brain parenchyma/CSF edge displacement between the two time points is then estimated by aligning the peaks of the spatial derivatives of the intensity profiles of both images. Finally, the mean edge displacement is converted into a global estimate of percentage brain volume change between the two time points, referred to as SIENA.

### Statistics

Based on the acquired MRI datasets, within scanner test–retest measurement errors for both 1.5T and 3T scanners, as well as the between scanner measurement errors are evaluated. For these experiments, the median over the patient population of the absolute values of the PBVC is calculated and denoted as the median percentage error. This is done for the PBVC of GM and PV obtained by MSmetrix and for the PBVC of PV obtained by SIENA. As these absolute values of the measurement errors are not normally distributed, the nonparametric paired Wilcoxon signed rank test was used to compare the errors between MSmetrix and SIENA for the within‐ and between‐scanner comparisons. In order to visually compare the results of MSmetrix and SIENA on the same datasets, Bland–Altman plots were generated for the measurement errors of both methods.

## Results

**Figure 2:**
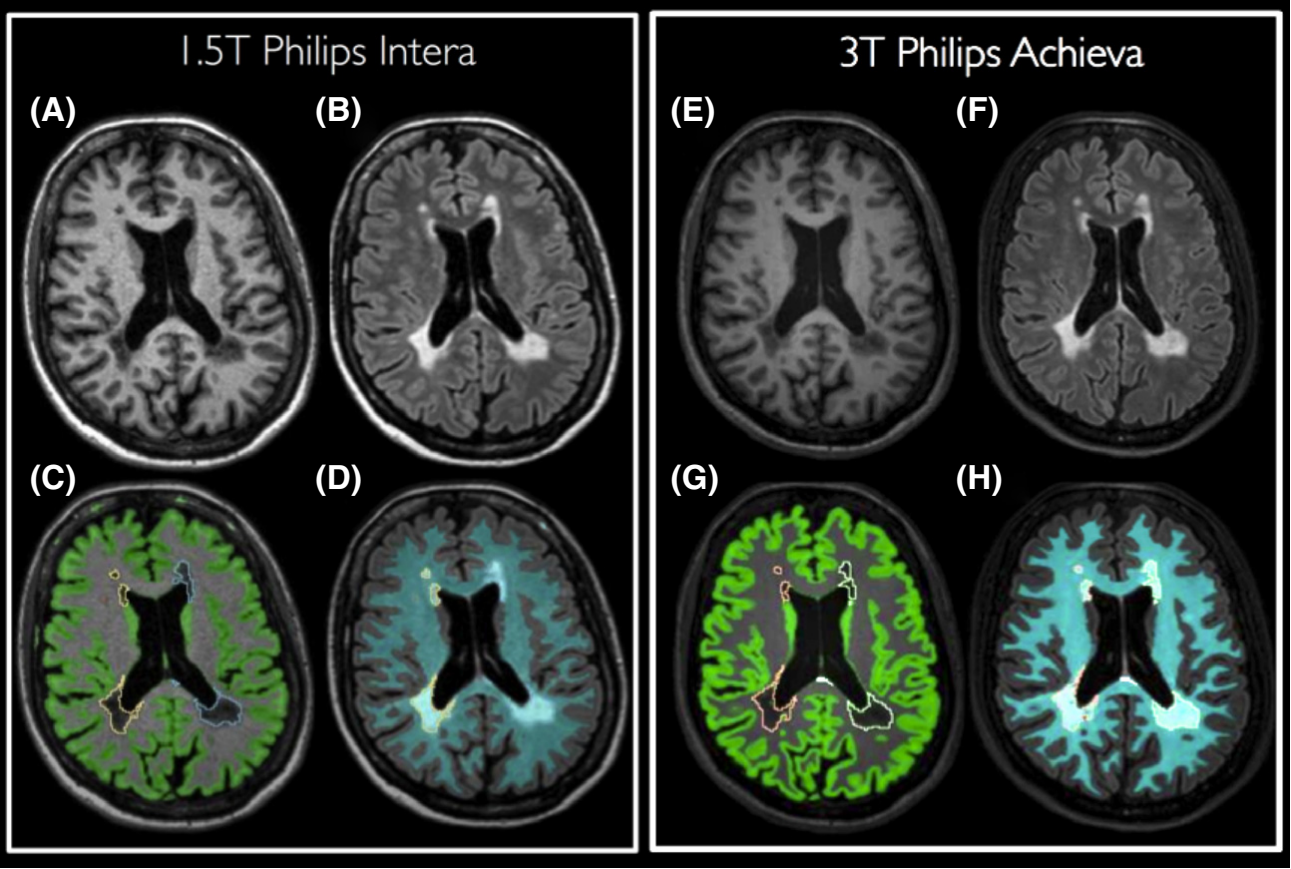
Visualization of MSmetrix segmentation results for the 1.5T (A–D) and 3T (E–H) scan of a randomly selected MS patient. T1 scans are shown in (A) and (E), FLAIR scans in (B) and (F). Lesion and GM segmentations are superimposed on the T1 scan, as displayed in (C) and (G). Finally, lesion and WM segmentations are visualized on the FLAIR scan in (D) and (H).

In Figure 2, some visual results of the MSmetrix segmentations on a 1.5T and 3T scan of the same randomly selected subject are displayed. In Figure 2A and B, an axial slice of the 1.5T 3D T1 and 3D FLAIR are shown, respectively. For visualization purposes, the GM and lesion segmentation are visualized on the T1 (c) and the WM and lesion masks on the FLAIR (d). A similar slice was selected for the 3T scan, as shown in Figure 2E and F, for the 3D T1 and 3D FLAIR, respectively. Similar as in Figure 2C and D, the GM, lesions, and WM segmentations of the 3T MRI are displayed in Figure 2G and H. These lesion segmentations are then used to fill the 3D T1 with normal‐appearing white matter, as explained in Figure 1. The cross‐sectional brain tissue segmentations that are shown in Figure 2 will be used as input for the longitudinal pipeline, to calculate the Jacobian of the deformation fields between both scans, resulting in a measure of brain and GM PBVC.

**Figure 3:**
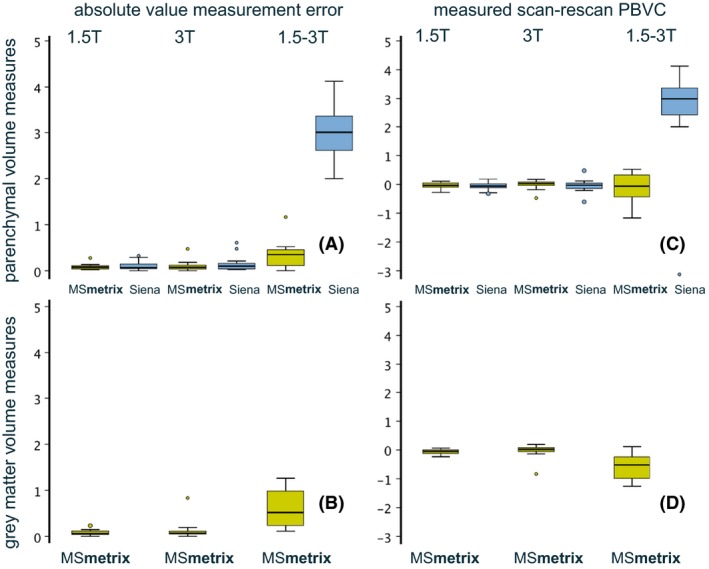
Boxplots of the measurement error. On the left, the boxplots of the absolute values of the measurement errors are shown for the parenchymal volume (A) and gray matter (B). On the right, boxplots of the measured scan–rescan PBVC (without taking the absolute value) are displayed for the parenchymal volume (C) and gray matter (D).

Boxplots of the measurement errors of the scan–rescan evaluations are presented in Figure 3. For the within scanner comparisons of the 1.5T and 3T scanner as well as the between‐scanner comparisons, boxplots of the absolute value of the measurement error (Fig. 3A and B) and of the measured scan–rescan PBVC (Fig. 3C and D) are displayed for both PV and GM. In Figure 3, MSmetrix results are shown in green, SIENA results in blue. The corresponding median and interquartile range of the absolute value of the measurement errors are displayed in Table 3.

**Table 3:** Median and interquartile range (IQR) of the intra and interscanner test–retest measurement errors for PV and GM (in %)

	1.5T Intera		3T Achieva		1.5T versus 3T	
	Median	IQR	Median	IQR	Median	IQR
GM MSmetrix	0.061	0.07	0.067	0.04	0.52	0.70
PV MSmetrix	0.078	0.06	0.071	0.08	0.35	0.32
PV Siena	0.065	0.09	0.100	0.12	2.99	0.85

PV, parenchymal volume; GM, gray matter.

In Table 4, the median of the calculated PBVC measures (without taking the absolute value) are shown. These numbers represent the potential bias to measuring negative or positive atrophy within and between scanners.

**Table 4:** Median and interquartile range (IQR) of the intra and interscanner PBVC measures for PV and GM (in %)

	1.5T Intera		3T Achieva		1.5T versus 3T	
	Median	IQR	Median	IQR	Median	IQR
GM MSmetrix	−0.047	0.10	0.013	0.12	−0.52	0.70
PV MSmetrix	−0.036	0.14	0.023	0.12	−0.061	0.72
PV Siena	−0.056	0.12	−0.033	0.19	2.99	0.85
Wilcoxon PV	0.47		0.078		0.0002	

PV, parenchymal volume; GM, gray matter; PBVC, percentage brain volume change.

**Figure 4:**
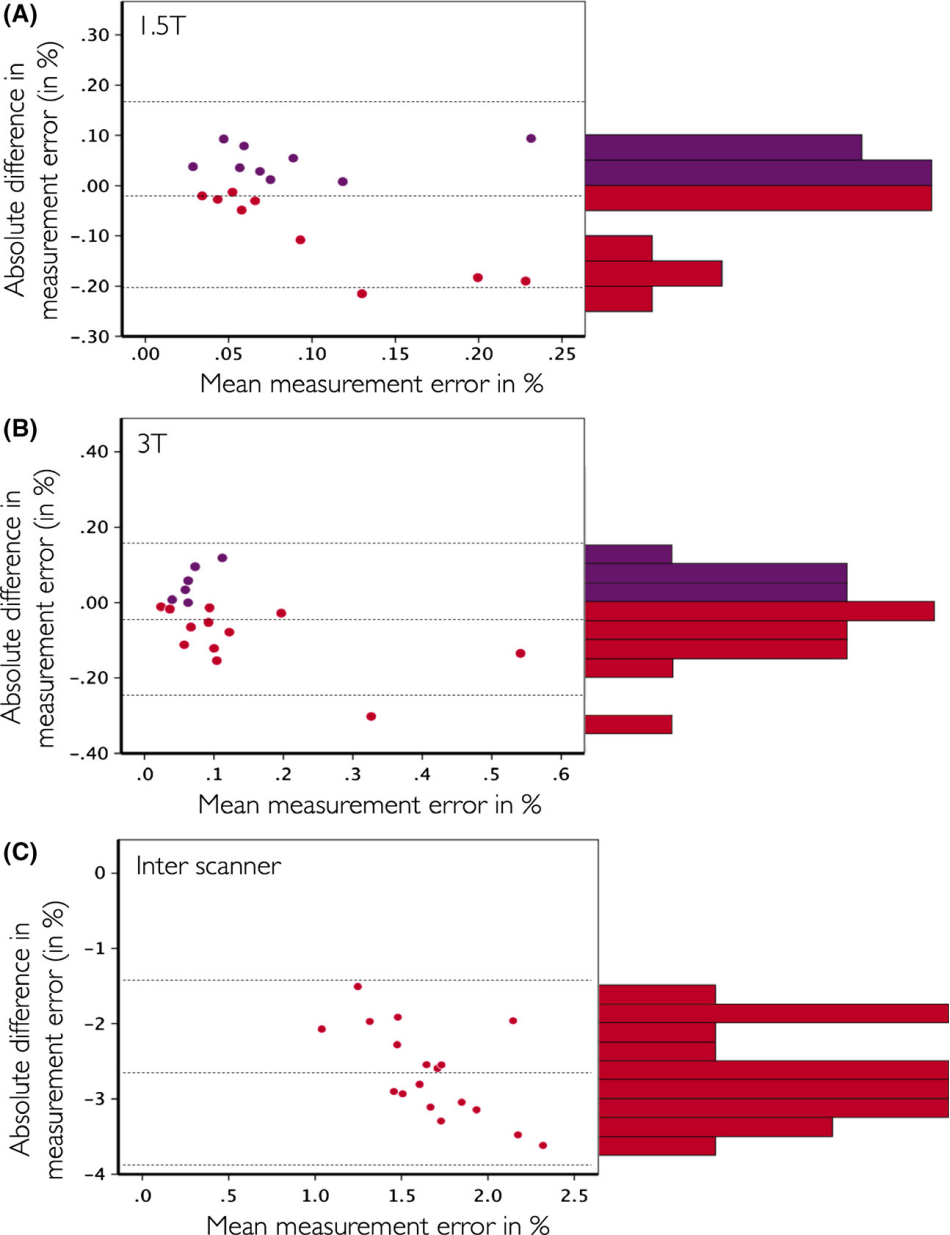
Bland–Altman plots of the comparison MSmetrix versus SIENA on the same datasets for the 1.5T within scanner (A), 3T within scanner (B), and 1.5T versus 3T between scanner (C) comparisons. On the *Y*‐axis of all plots, the difference of the absolute value of the measurement errors is calculated as ‘MSmetrix – SIENA’, on the *X*‐axis of all plots the mean of MSmetrix and SIENA is displayed. Purple dots were used when ‘MSmetrix – SIENA’ is positive, red dots when this difference is negative. In addition, the histogram of the ‘MSmetrix – SIENA’ difference is shown on the right of each Bland–Altman plot.

In Figure 4, the Bland–Altman plots of the absolute value of the measurement error are displayed for the intrascanner comparison at 1.5T (Fig. 4A), the intrascanner comparison at 3T (Fig. 4B), and the between scanner (1.5T vs. 3T) comparison (Fig. 4C). As the difference of the absolute measurement error for ‘MSmetrix – SIENA’ is calculated, a positive difference indicates a smaller error for SIENA compared to MSmetrix (purple dots) and a negative difference presents a smaller error for MSmetrix compared to SIENA (red dots). In addition, a histogram of the MSmetrix‐SIENA difference for the absolute value of the measure error is shown at the right side of each Bland–Altman plot.

## Discussion

Brain atrophy is a part of MS pathophysiology and is correlated with clinical outcomes, both physical and cognitive. Therefore, there is a need for measuring brain volume, and especially brain atrophy, in clinical practice for individual MS patients. In this manuscript, a longitudinal, Jacobian based method for measuring whole brain and gray matter atrophy is used. One of the main challenges of translating methods for brain atrophy from research analyses on groups of subjects to clinical practice in an individual patient is minimizing the measurement error of the assessment. To this end, in order to assess the use of this method in clinical practice on MRI datasets of individual MS patients, the measurement error of whole brain and gray matter volume measurements was evaluated in this manuscript. Results were compared to SIENA, a well‐validated method for measuring brain atrophy. Note that only whole‐brain volume results are compared with SIENA, as no gray matter volume is measured with this software. To evaluate the measurement error of the brain volume measurement software packages, two sets of MRI data from a 1.5T and a 3T MRI scanner were acquired in 19 MS patients on the same day. It is then assumed that the brain volume would be the same between all MRI exams of each individual MS patient. The MRI protocol on each scanner consisted of a standard, nonoptimized or harmonized 3D T1 and a 3D FLAIR. We notice that SIENA shows a large bias due to contrast differences. Volumes are consistently bigger when measured on a 3T image compared to a 1.5T image. MSmetrix is more robust to these contrast differences due to regularization, where the whole brain is considered to determine the atrophy and not only the borders.

The MSmetrix software pipeline is specifically designed to measure atrophy in patients with MS, by including iterative lesion segmentation and lesion filling based on FLAIR and T1‐weighted MRI scans. In this context, it is known that applying brain atrophy measures without performing lesion filling can introduce errors between 0.3% and 2.5%, depending on the lesion size and lesion intensity (Chard et al. 2010; Battaglini et al. 2012; Popescu et al. 2014). As all MRI scans were acquired on the same day, no changes in lesion volume or distribution are expected in the data that were analyzed. Performing lesion filling before the volume measures did not have an effect on the presented results and no additional errors have been added to the errors mentioned in this manuscript.

To the best of our knowledge, this is the first paper describing measurement errors of brain atrophy methods based on scan–rescan MRI datasets from different scanners on patients with MS. Other studies already evaluated scan–rescan errors in healthy subjects or patients with dementia (Smith et al. 2001a,b; Cover et al. 2014; Nakamura et al. 2014). Another difference with these studies is that the MRI datasets used in our study were acquired using a clinical MRI protocol with 3D sequences. No optimized and typically longer research sequences were used, and the T1 and FLAIR sequences were not optimized within each scanner or harmonized between both scanners. In this context, in order to introduce brain atrophy measures in clinical practice, they should have an acceptable measurement error on MRI scans that can be obtained in a clinical setting with a limited acquisition time. As a result, the reproducibility results presented in this paper can be seen as representative for a clinical setting for patients with MS.

Our results demonstrate that a small brain volume measurement error can be achieved, especially when data of the same scanner are compared, in the order of 0.06–0.08% for both MSmetrix and SIENA. However, it should be noted that in this study, patients were not removed from the scanner in between both acquisitions on the same scanner. As a result, for the intrascanner comparison, patients were positioned in the same way, which did not affect the measurement error results. This can explain the lower measurement errors that were reported here for SIENA, compared to previously published studies, where errors in the order of 0.2% were found (Smith et al. 2001a,b). Obviously, on the different scanners, patients were repositioned. Due to the repositioning, different sequences, different contrasts, the measurement errors were larger when scans from 1.5T and 3T were compared. Especially for SIENA, a significant larger measurement error was observed for the between‐scanner analysis. In addition to an increased absolute error, it can be observed that a large bias was found. Although a trend was observed of a smaller measurement error for MSmetrix compared to SIENA for the within‐scanner tests, only for the between‐scanner comparison the Wilcoxon signed rank test indicated a significant difference. In contrast to SIENA, MSmetrix is able to also measure GM atrophy using a longitudinal approach.

Our study has other limitations. First, a small cohort of patients was included (18). Second, it is important to notice that all scans were acquired on Philips systems. Further research is needed to evaluate brain volume measurement errors on other MRI scanners. In conclusion, results of this study provide insights in the difference between 1.5T and 3T scanners and the clinical usability of automated measures on both scanner types. MSmetrix appeared robust on both the 1.5T and 3T systems, where it should be noted that the measurement error becomes an order of magnitude higher between scanners with different field strength.

## Conflict of Interest

None declared.
